# DNMT3L facilitates DNA methylation partly by maintaining DNMT3A stability in mouse embryonic stem cells

**DOI:** 10.1093/nar/gky947

**Published:** 2018-10-13

**Authors:** Nicolas Veland, Yue Lu, Swanand Hardikar, Sally Gaddis, Yang Zeng, Bigang Liu, Marcos R Estecio, Yoko Takata, Kevin Lin, Mary W Tomida, Jianjun Shen, Debapriya Saha, Humaira Gowher, Hongbo Zhao, Taiping Chen

**Affiliations:** 1 Department of Epigenetics and Molecular Carcinogenesis, The University of Texas MD Anderson Cancer Center, Smithville, TX 78957, USA; 2 Center for Cancer Epigenetics, The University of Texas MD Anderson Cancer Center, Smithville, TX 78957, USA; 3 Program in Genetics and Epigenetics, The University of Texas MD Anderson Cancer Center UTHealth Graduate School of Biomedical Sciences, Houston, TX 77030, USA; 4 Department of Biochemistry, Purdue University, West Lafayette, IN 47907, USA; 5 Purdue University Center for Cancer Research, Purdue University, West Lafayette, IN 47907, USA; 6 Shanghai Key Laboratory of Female Reproductive Endocrine Related Diseases, Hospital and Institute of Obstetrics and Gynecology, Fudan University, Shanghai, People's Republic of China

## Abstract

DNMT3L (DNMT3-like), a member of the DNMT3 family, has no DNA methyltransferase activity but regulates *de novo* DNA methylation. While biochemical studies show that DNMT3L is capable of interacting with both DNMT3A and DNMT3B and stimulating their enzymatic activities, genetic evidence suggests that DNMT3L is essential for DNMT3A-mediated *de novo* methylation in germ cells but is dispensable for *de novo* methylation during embryogenesis, which is mainly mediated by DNMT3B. How DNMT3L regulates DNA methylation and what determines its functional specificity are not well understood. Here we show that DNMT3L-deficient mouse embryonic stem cells (mESCs) exhibit downregulation of DNMT3A, especially DNMT3A2, the predominant DNMT3A isoform in mESCs. DNA methylation analysis of DNMT3L-deficient mESCs reveals hypomethylation at many DNMT3A target regions. These results confirm that DNMT3L is a positive regulator of DNA methylation, contrary to a previous report that, in mESCs, DNMT3L regulates DNA methylation positively or negatively, depending on genomic regions. Mechanistically, DNMT3L forms a complex with DNMT3A2 and prevents DNMT3A2 from being degraded. Restoring the DNMT3A protein level in DNMT3L-deficient mESCs partially recovers DNA methylation. Thus, our work uncovers a role for DNMT3L in maintaining DNMT3A stability, which contributes to the effect of DNMT3L on DNMT3A-dependent DNA methylation.

## INTRODUCTION

DNA methylation—the addition of a methyl group to the C-5 position of cytosine, forming 5-methylcytosine (5mC)—occurs predominantly in the context of CpG dinucleotides in mammals. DNA methylation is essential for mammalian development and plays crucial roles in various biological processes, including regulation of gene expression, maintenance of genomic stability, genomic imprinting, and X chromosome inactivation (1,2). Aberrant DNA methylation patterns and genetic alterations of the DNA methylation machinery are associated with numerous human diseases, including developmental disorders and cancer (3,4).

DNA methylation is catalyzed by two classes of DNA methyltransferases (DNMTs). DNMT1 is the major enzyme responsible for maintenance methylation by ‘copying’ the CpG methylation pattern from the parental strand onto the daughter strand during DNA replication. DNMT3A and DNMT3B function primarily as *de novo* methyltransferases for the establishment of DNA methylation patterns during embryogenesis and gametogenesis (2,5). *Dnmt3c*, a *Dnmt3b* duplicated gene present exclusively in rodents, had been previously annotated as a pseudogene but was recently shown to be expressed and play a specific role in repressing retrotransposons during spermatogenesis (6). The DNMT1 and DNMT3 enzymes share characteristic catalytic motifs in their C-terminal catalytic domains but have distinct N-terminal regulatory regions that contribute to the functional specificities of these enzymes (5). For example, DNMT3A and DNMT3B contain two chromatin-binding domains in their N-terminal regions that likely play important roles in targeting these enzymes to specific genomic regions—the PWWP domain, which is required for heterochromatin targeting and mediates binding to histone H3 lysine 36 trimethyl (H3K36me3) marks (7–9), and the ADD domain, which specifically recognizes the N-terminal tail of histone H3 when lysine 4 is unmodified (H3K4me0) (10). There is evidence that, in mouse embryonic stem cells (mESCs), DNMT3B is targeted to gene bodies via PWWP domain-H3K36me3 interaction and, upon mESC differentiation, DNMT3A is specifically targeted to the enhancers of pluripotency genes via ADD domain-H3K4me0 interaction to silence these genes (9,11). Although DNMT3A and DNMT3B redundantly methylate many genomic regions, they also have preferred and specific DNA targets. For example, DNMT3A preferentially methylates the major satellite repeats, and DNMT3B preferentially methylates the minor satellite repeats (12). Target specificities of these enzymes likely contribute, to a great extent, to their distinct functions. *Dnmt3a* knockout (KO) mice develop to term and die postnatally, and mice with conditional deletion of *Dnmt3a* in the germline fail to undergo *de novo* methylation during gametogenesis, including the establishment of methylation imprints, resulting in spermatogenesis defects and maternal-effect lethality—embryos derived from *Dnmt3a* KO females die around mid-gestation (13,14). *Dnmt3b* KO mice are embryonically lethal (13).

DNMT3L (DNMT3-like), another member of the DNMT3 family, shows sequence homology with the DNMT3A/3B enzymes but lacks the very N-terminal region, including the PWWP domain, and some essential catalytic motifs in the C-terminal region, including the PC dipeptide at the active site and the sequence motif involved in binding the methyl donor S-adenosyl-L-methioinine. Thus, DNMT3L has no DNA methyltransferase activity (15–17). However, DNMT3L has been shown to interact with DNMT3A and DNMT3B and significantly stimulates their catalytic activities *in vitro* (18–23). Crystallography evidence reveals that the C-terminal domain of DNMT3L directly interacts with the C-terminal domain of DNMT3A to form a heterodimer, which further dimerizes through DNMT3A-DNMT3A interaction, resulting in the formation of a tetramer with two DNMT3A active sites at the center (24–26). Biochemical and structural data also indicate that DNMT3L, via its ADD domain, interacts with the N-terminal tail of histone H3 with unmethylated lysine 4 (H3K4me0) and may contribute to the specificity of *de novo* methylation (27). Additionally, the expression pattern of DNMT3L during development strongly correlates with active *de novo* methylation. Specifically, *Dnmt3l* is highly expressed in developing germ cells and early embryos, as well as mESCs, but is silenced in somatic tissues (17). These findings suggest that DNMT3L is a key regulator of *de novo* DNA methylation.

Genetic evidence supports the role of DNMT3L in the regulation of *de novo* methylation. *Dnmt3l* KO mice are viable and grossly normal, indicating that zygotic DNMT3L is not essential for embryonic development. However, both male and female KO mice cannot reproduce (17,28). Male KO mice show activation of retrotransposons in spermatogonia and spermatocytes, due to failure in *de novo* DNA methylation, resulting in spermatogenesis defects (29). Female KO mice show a severe deficiency in *de novo* DNA methylation during oogenesis, including the establishment of maternal genomic imprints. These animals show a maternal-effect lethality phenotype, which correlates with abnormal expression of imprinted genes (17,28). In summary, the germline phenotype of *Dnmt3l* KO mice is almost identical to that of mice with conditional *Dnmt3a* deletion, whereas deletion of zygotic *Dnmt3l* results in no overt abnormalities in embryonic development and adult mice, in contrast to the embryonic lethal phenotype of *Dnmt3b* KO mice (13,14).

Although it is generally believed that DNMT3L acts as an accessory factor of DNMT3A and DNMT3B, the mechanism by which DNMT3L regulates DNA methylation is not fully understood. In particular, given that DNMT3L is capable of interacting with and enhancing the activities of both DNMT3A and DNMT3B (18–23), it has been puzzling that DNMT3L is essential for DNMT3A-mediated *de novo* methylation during gametogenesis but is dispensable for DNMT3B-mediated *de novo* methylation during embryogenesis (13,14,17,28). Moreover, contrary to biochemical and genetic evidence that DNMT3L functions as a positive regulator of DNA methylation, Neri *et al.* showed that DNMT3L antagonizes DNA methylation at bivalent promoters [promoters enriched with both the active histone H3 lysine 4 trimethyl (H3K4me3) and the repressive lysine 27 trimethyl (H3K27me3) marks] and favors DNA methylation at gene bodies in mESCs (30). To gain further insights into the role and specificity of DNMT3L in DNA methylation, we derived mESC lines deficient for maternal DNMT3L (*Dnmt3l* mKO), zygotic DNMT3L (*Dnmt3l* zKO), or both maternal and zygotic DNMT3L (*Dnmt3l* mzKO) from blastocyst-stage embryos. We confirmed that DNMT3L is a positive regulator of DNA methylation and found no evidence that it antagonizes DNA methylation in mESCs. While *Dnmt3l* mKO mainly affected DNA methylation at imprinting control regions (ICRs), *Dnmt3l* zKO mESC lines showed moderate loss of methylation at many genomic regions that are methylated by DNMT3A. Interestingly, our results showed that DNMT3L is critical for maintaining the stability of DNMT3A, especially DNMT3A2, the predominant DNMT3A protein product in mESCs, and that this new role contributes to the specific effect of DNMT3L on DNMT3A-dependent DNA methylation.

## MATERIALS AND METHODS

### Mice

The *Dnmt3l* null allele, generated previously (17), was maintained on the 129 background. All procedures were performed according to the National Institutes of Health Guide for the Care and Use of Laboratory Animals, with Institutional Care and Use Committee-approved protocols at The University of Texas MD Anderson Cancer Center (MDACC).

### mESC derivation, culture and treatments

Derivation of mESC lines was performed as previously described (31). Briefly, female mice were euthanized at 3.5 days post coitum, and blastocysts were flushed out of the uterus using M2 medium (Sigma). Then, blastocysts were placed on a feeder cell layer of irradiated mouse embryonic fibroblasts (MEFs) in mESC medium [DMEM supplemented with 15% fetal bovine serum, 0.1 mM nonessential amino acids, 0.1 mM β-mercaptoethanol, 50 U/ml penicillin, 50 μg/ml streptomycin, and 10^3^ U/ml leukemia inhibitory factor (LIF)]. After blastocyst hatching, the inner cell mass (ICM) clumps were picked under the microscope and plated into 96-well plate with feeder cells. The established mESC lines were genotyped (to determine *Dnmt3l* allele and sex) and cryopreserved.

The mESC lines were cultured on gelatin-coated petri dishes in serum-containing mESC medium. Normally, the medium was changed daily, the cells were passaged every other day, and the passage numbers were recorded. For the generation of stable mESC clones expressing Myc-DNMT3L, -DNMT3L F297D, -DNMT3A1 or -DNMT3A2, cells transfected with the corresponding plasmids were seeded at low density on dishes coated with feeder cells, selected with 6 μg/ml of Blasticidin S HCl (Gibco) for 7–10 days, and individual colonies were picked. *Dnmt3l* knockdown (KD) mESCs were generated using the same plasmid vectors and protocols reported by Neri *et al.* (30). Briefly, J1 mESCs were transfected with vectors encoding *Dnmt3l* shRNAs (*shDnmt3l* #1: TRCN0000039104; *shDnmt3l* #2: TRCN0000039106; *shDnmt3l* #3: TRCN0000039108) or a *luciferase* shRNA (*shLuc*) as control, followed by three days of selection with 1 μg/ml of Puromycin. Transfection was performed using Lipofectamine 2000 (Invitrogen). For some experiments, mESCs were treated with cycloheximide (C4859, Sigma) at 100 μg/ml, MG132 (M7449, Sigma) at 10 μM, or Bafilomycin A1 (B1793, Sigma) at 125 nM, with DMSO as a negative control. ESC differentiation was induced by LIF withdrawal followed by retinoic acid addition on day 3 post-differentiation (32).

### Southern blot analysis

DNA methylation levels at the major and minor satellite repeats were analyzed by Southern blot after digestion of 1 μg of genomic DNA with the methylation-sensitive restriction enzymes MaeII (Roche) or HpaII (New England Biolabs), respectively, as previously reported (33). Detection was performed using biotin-labelled probes (300 ng each) and the North2South Chemiluminescent Hybridization and Detection Kit (Thermo Fisher Scientific). Probe sequences are shown in Supplementary Table S1.

### Bisulfite sequencing

Bisulfite sequencing analysis of the DNA methylation levels at specific regions was performed as previously described (33). Briefly, genomic DNA was treated for bisulfite conversion using the EZ DNA Methylation kit (Zymo Research) and then used as template to amplify regions of the *Rhox5, Cpne8, Enox1, Zxda* and *Hoxa1* loci using specific primer pairs (Supplementary Table S1). PCR products were gel purified and directly ligated into pMiniT 2.0 using the NEB PCR cloning kit (New England Biolabs). Miniprep DNA was sequenced and results were analyzed with QUMA web-based quantification tool for methylation analysis (http://quma.cdb.riken.jp). Percentages of methylated CpG sites were calculated based on results from multiple clones.

### RNA isolation and RT-qPCR

Total RNA was extracted from mESCs using TRIzol (Invitrogen) according to the manufacturer's instruction. Reverse transcription (RT) was performed using ProtoScript First Strand cDNA Synthesis kit (New England Biolabs) to generate cDNA. RT-qPCR was performed using iTaq Universal SYBR Green Supermix (Bio-Rad) on the ABI 7900 Real-Time PCR system (Thermo Fisher Scientific) using specific primers (Supplementary Table S1).

### Western blot and immunoprecipitation

For analysis of whole cell extracts by western blot, mESCs were lysed in cold RIPA buffer [50 mM Tris–HCl (pH 8.8), 150 mM NaCl, 1% Triton X-100, 0.5% Sodium Deoxycholate, 0.1% SDS, 1 mM EDTA, 3 mM MgCl_2_, and 1× protease inhibitor cocktail (Thermo Fisher Scientific)]. Immunoprecipitation (IP) was performed in lysis buffer [20 mM Tris–HCl (pH 7.9), 150 mM NaCl, 0.1% NP-40, 1 mM EDTA, 3 mM MgCl_2_, 10% glycerol and 1× protease inhibitor cocktail (Thermo Scientific)] using Protein A/G UltraLink Resin beads (Thermo Fisher Scientific). Western blot was performed according to standard procedures. The antibodies used are listed in Supplementary Table S2. Quantification of western blots by densitometry, normalized against β-ACTIN, was carried out using NIH ImageJ software (34).

### Immunofluorescence analysis

Zygotes were fixed in 3.7% paraformaldehyde in PBS for 30 min at room temperature and permeabilized for 15 min in 0.1% Triton X-100 in PBS at room temperature. 5mC and 5-hydroxymethylcytosine (5hmC) staining was carried out as previously described (33). The antibody information is provided in Supplementary Table S2. Images were taken with Olympus IX51 inverted fluorescence microscope.

### Reduced representation bisulfite sequencing (RRBS) and bioinformatics analyses

RRBS libraries were made from 1 μg of genomic DNA, according to published protocols (35,36). In brief, genomic DNA was digested with MspI, end-repaired and A-tailed, and Illumina-compatible cytosine-methylated adaptor (Bioo Scientific) was ligated to the enzyme-digested DNA. Size-selected fragments were bisulfite-converted using the EZ DNA Methylation-Gold kit (Zymo Research), and library preparation was done by PCR amplification. The libraries were sequenced using 36 bp single-read protocol on Illumina HiSeq 2500 instrument.

Three biological replicates were prepared for each genotype (one *Dnmt3l* zKO sample was discarded in later analysis due to poor quality). 12–35 million reads were generated per sample. The adapters were removed from the 3′ ends of the reads by Trim Galore! version 0.4.1 (https://www.bioinformatics.babraham.ac.uk/projects/trim_galore/) and cutadapt version 1.9.1 (37). Then, the reads were mapped to mouse genome mm10 by the bisulfite converted read mapper Bismark version 0.16.1 (38) and Bowtie version 1.1.2 (39). 95–96% reads were mapped to the mouse genome, with 65–68% uniquely mapped. 8–23 million uniquely mapped reads were used in the final analysis. The methylation percentages for the CpG sites were calculated by the bismark_methylation_extractor script from Bismark and an in-house Perl script. The differential methylation between genotypes was statistically assessed by R/Bioconductor package methylKit version 0.9.5 (40) at site resolution. RRBS was tiled (500 bp) when comparing with the whole genome bisulfite sequencing (WGBS) data of *Dnmt3a* KO and *Dnmt3b* KO mESCs. Only the CpG sites with read coverage ≥20 or the tiles that have at least three CpG sites with coverage ≥10 in all the samples were qualified for the test. The CpG sites or 500-bp tiles with *q*-value ≤0.01 and methylation difference ≥25% were called as differentially methylated. Each site or tile was assigned to a location relative to the nearby genes: upstream (−5k to −1k from TSS), promoter (−1k to +0.5k from TSS), exon, intron, TES (−0.5k to +1k from TES), downstream (+1k to +5k from TES) and intergenic. In the case a site/tile could be assigned to multiple locations relative to different genes, one location was chosen following this order: promoter > upstream > TES > downstream > exon > intron > intergenic. The genes are the RefSeq genes (41) downloaded from UCSC genome browser (http://genome.ucsc.edu/) on 17 July 2015.

## RESULTS

### DNMT3L deficiency in mESCs results in hypomethylation at specific heterochromatin regions

**Figure 1.**
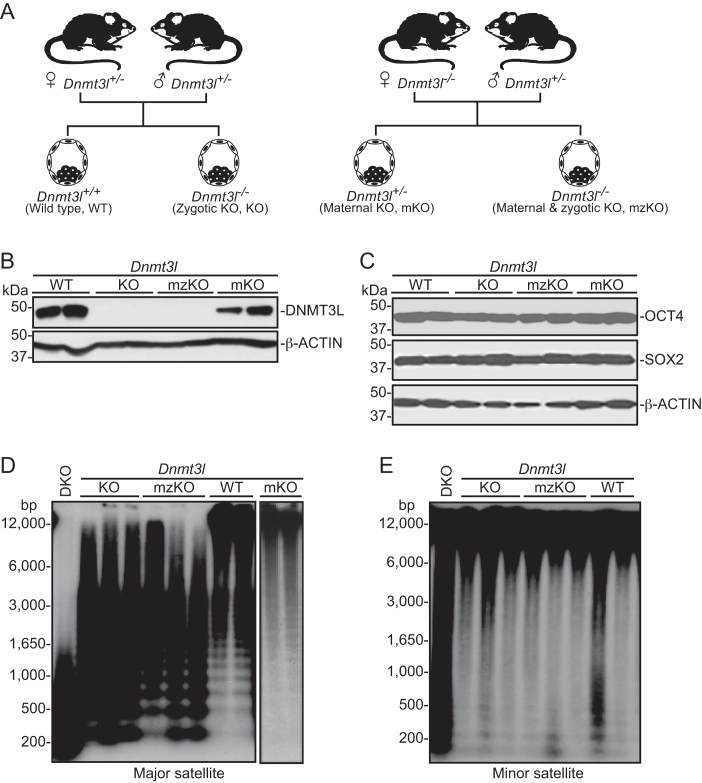
DNMT3L deficiency in mESCs results in hypomethylation at specific heterochromatin regions. (**A**) Mouse breeding strategies to generate mESC lines with different *Dnmt3l* genotypes. (**B** and **C**) Western blot analysis of the mESC lines (2 independent clones per genotype) for expression of DNMT3L (B) and the pluripotency factors OCT4 and SOX2 (C), with β-ACTIN serving as a loading control. (**D** and **E**) Southern blot analysis of the mESC lines (2-3 independent clones per genotype) for DNA methylation at the major satellite repeats (D) and the minor satellite repeats (E) after digestion of genomic DNA with methylation-sensitive restriction enzymes (MaeII for major satellite repeats and HpaII for minor satellite repeats). DKO, *Dnmt3a/3b* double KO mESC line.

Genetic studies in mouse have demonstrated that maternal DNMT3L is critical for DNA methylation in oocytes, including the establishment of genomic imprints, whereas zygotic DNMT3L is not essential for mammalian development (17,28,42,43). To better understand the roles of maternal and zygotic DNMT3L in DNA methylation, we derived mESC lines deficient for maternal DNMT3L (*Dnmt3l* mKO), zygotic DNMT3L (*Dnmt3l* zKO, referred to as *Dnmt3l* KO hereafter), or both maternal and zygotic DNMT3L (*Dnmt3l* mzKO), as well as WT (*Dnmt3l*^+/+^) mESC lines, from blastocyst-stage embryos by breeding *Dnmt3l* homozygous (*Dnmt3l^−/−^*) or heterozygous (*Dnmt3l*^+/−^) females with *Dnmt3l*^+/−^ males (Figure 1A). As female mESCs become hypomethylated in culture due to upregulation of DUSP9, an X-linked MAPK phosphatase (44), and DNMT3L deficiency in mESCs, especially in female mESCs, results in progressive loss of methylation (45), we used male mESC lines (determined by PCR amplification of Y-linked *Sry*) that had been cultured for 10–15 passages for all experiments. The mESC lines were genotyped by PCR and verified by western blot analysis (Figure 1B). All *Dnmt3l*-mutant cell lines maintained the mESC state, as judged by colony morphology, growth rates, and expression of the pluripotency factors OCT4 and SOX2 (Figure 1C).

In general, repetitive sequences in mammalian genomes are heavily methylated, and their methylation status can serve as an indicator of global DNA methylation. To assess the impact of DNMT3L deficiency on DNA methylation, we first compared the different mESC lines for DNA methylation at the major and minor satellite repeats, which are located at pericentric and centromeric regions, respectively. Southern blot analysis of genomic DNA digested with methylation-sensitive restriction enzymes revealed that *Dnmt3l* KO and *Dnmt3l* mzKO mESC lines had substantial loss of methylation at the major satellite repeats, but no obvious changes in methylation at the minor satellite repeats (Figure 1D, E). Consistent with previous reports that maternal DNMT3L is required for global DNA methylation in oocytes (42,43), the female pronuclei of zygotes derived from *Dnmt3l^−/-^*oocytes were severely hypomethylated (Supplementary Figure S1). However, *Dnmt3l* mKO mESC lines, unlike *Dnmt3l* KO and *Dnmt3l* mzKO cells, showed normal levels of DNA methylation at the major satellite repeats (Figure 1D), indicating that *de novo* methylation had occurred either during preimplantation development of *Dnmt3l* mKO embryos or during mESC derivation and culture, leading to recovery of global DNA methylation levels (except at imprinted loci, see below). Our results show that DNMT3L deficiency affects DNA methylation at specific genomic regions in mESCs. Consistent with previous reports that hypomethylation impairs mESC differentiation (31,46). *Dnmt3l* KO mESCs exhibited obvious defects in forming embryoid bodies (EBs) under differentiation conditions, with EBs being small, irregular and fragmented (Supplementary Figure S2).

### DNMT3L is a positive regulator of DNA methylation in mESCs

**Figure 2.**
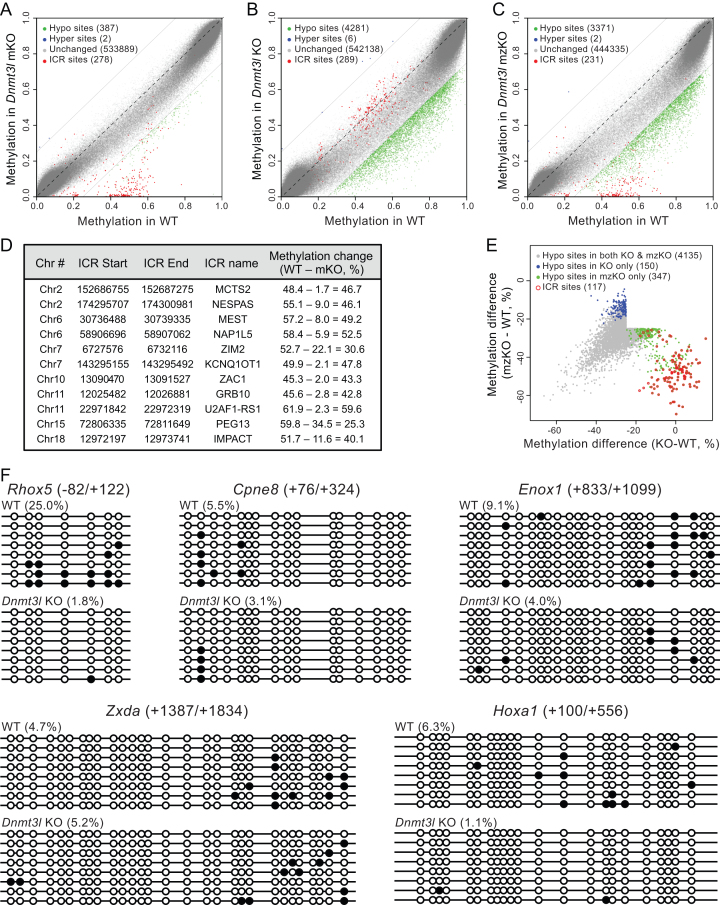
DNMT3L is a positive regulator of DNA methylation in mESCs. (A–C) Scatter plots of RRBS data in *Dnmt3l* mKO (**A**), KO (**B**) and mzKO (**C**) mESCs compared to WT mESCs. Each dot represents a CpG site. CpG sites within known ICRs are shown in red. For other CpG sites, significantly (methylation difference ≥25% and *q*-value ≤ 0.01) hypomethylated ones are shown in green, hypermethylated ones in blue, and those with methylation changes of <25% in gray. (**D**) ICRs with significantly hypomethylated sites in *Dnmt3l* mKO mESCs. The start and end locations of the ICRs were from WAMIDEX (http://atlas.genetics.lcl.ac.uk) with minor revisions to the boundaries of the ZAC1 and GRB10 ICRs based on our data. Specifically, two previously annotated ZAC1 ICRs separated by 346 bp were considered a continuous ICR, as 34 of 35 hypomethylated CpGs in *Dnmt3l* mKO mESCs fell between the regions, and the GRB10 ICR was extended by 549 bp, as 26 of 58 hypomethylated CpGs in *Dnmt3l* mKO mESCs were 13–549 bp downstream of the end of the previously annotated ICR. (**E**) Scatter plot of the hypomethylated sites in *Dnmt3l* KO and mzKO mESCs showing overlap of the vast majority of these sites (gray) with the exception of those in ICRs (red), which were severely hypomethylated in *Dnmt3l* mzKO cells but showed no change in methylation in *Dnmt3l* KO cells. The plot was generated using the union of hypomethylated sites at methylation difference ≥25% and *q*-value ≤0.01 in *Dnmt3l* mzKO or *Dnmt3l* KO cells, with sites with *q*-value ≤0.01 in both genotypes being considered common sites. (**F**) Bisulfite sequencing analysis of five gene regions that Neri and colleagues showed gain of methylation in *Dnmt3l* KD mESCs, which revealed loss of or no changes in methylation in *Dnmt3l* KO mESCs instead. The beginning and end of each amplified region, relative to the transcription start site (TSS), are indicated. Open and filled circles represent unmethylated and methylated CpG sites, respectively. The methylation levels (in percentages) of each region in WT and *Dnmt3l* KO mESCs are shown.

Contrary to biochemical, genetic and genomic evidence that DNMT3L is a positive regulator of DNA methylation (17–19,21,27,28,42,43), Neri *et al.* reported that DNMT3L regulates DNA methylation positively or negatively, depending on genomic regions, in mESCs (30). To further understand the function and specificity of DNMT3L in DNA methylation, we compared the DNA methylation profiles of the different mESC lines (*Dnmt3l^+/+^, Dnmt3l* KO, *Dnmt3l* mKO, and *Dnmt3l* mzKO, 2–3 lines per genotype) by RRBS analysis (47–49). For each sample, we generated ∼12–35 million high quality RRBS reads, of which ∼8–23 million were uniquely mapped, capturing ∼454-679 thousand CpG sites with ≥ 20-fold coverage. Reads from all samples showed near complete (>99%) bisulfite conversion of cytosines in non-CpG contexts (Supplementary Table S3). In agreement with previous work (48), the methylation levels of CpG sites displayed a bimodal distribution in WT mESCs, as well as in the *Dnmt3l*-mutant cell lines, with most sites being either largely unmethylated (<20% methylation) or largely methylated (>80% methylation) (Figure 2A–C, Supplementary Figure S3).

Compared to WT mESCs, the *Dnmt3l*-mutant cell lines showed different degrees and distinct patterns of hypomethylation (Figure 2A–C). For the purpose of bioinformatics analysis, we defined differentially methylated CpG sites as those with methylation difference ≥25% and *q*-value ≤0.01. In *Dnmt3l* mKO cells, while the vast majority of CpG sites showed no significant methylation changes, many CpG sites within the ICRs of 11 maternally imprinted loci were severely hypomethylated (Figure 2A, D). These sites showed methylation levels ∼50% in WT cells (Figure 2A, D), consistent with monoallelic methylation of ICRs. In *Dnmt3l* KO cells, a considerable fraction of CpG sites in the genome (especially partially methylated sites) showed reduced methylation levels, mostly with moderate decreases, resulting in an obvious trend of hypomethylation (Figure 2B). Specifically, ∼8.5% (3074 of 36 198) of partially (20-80%) methylated and ∼1.1% (1207 of 108 956) of highly (≥80%) methylated CpG sites in WT cells were significantly hypomethylated in *Dnmt3l* KO cells. Notably, CpG sites in ICRs were completely resistant to hypomethylation in these cells (Figure 2B). *Dnmt3l* mzKO cells exhibited combined changes of both *Dnmt3l* mKO and *Dnmt3l* KO cells, with severe loss of methylation in CpG sites in ICRs and moderate hypomethylation of many sites in other regions (Figure 2C, E). Taken together, our results indicate that maternal DNMT3L is essential for the establishment of germline imprints, consistent with previous studies (17,28), but has no major effect on global DNA methylation in mESCs, whereas expression of zygotic DNMT3L is important for the methylation levels of a subset of genomic regions, but not for the maintenance of DNA methylation at imprinted loci, in mESCs.

In sharp contrast to the report by Neri *et al.* that nearly 30% of the differentially methylated regions (DMRs) show gain of methylation in shRNA-mediated *Dnmt3l* KD mESCs (30), we observed only negligible numbers of hypermethylated CpG sites in the *Dnmt3l*-mutant mESC lines (Figure 2A–C). Given that Neri and colleagues showed that DNMT3L deficiency-induced gain of methylation occurred mostly at bivalent promoter regions, it is unlikely that the discrepancy was due to limited coverage of the genome by RRBS, as RRBS preferentially captures CG-rich sequences including many CpG islands and promoters. Indeed, our RRBS data had good coverage of promoter regions (Supplementary Table S3). Neri and colleagues verified, using bisulfite sequencing analysis, five gene regions (*Rhox5, Cpne8, Enox1, Zxda* and *Hoxa1*) that showed gain of methylation in *Dnmt3l* KD cells (30). We searched our RRBS data for these regions and found that seven of the 22 CpG sites in the *Enox1* locus analyzed by Neri *et al.* had sufficient coverage (≥20) in both WT and *Dnmt3l* KO samples. However, our data revealed that all the seven sites had lower levels of methylation in *Dnmt3l* KO cells than in WT cells (Supplementary Figure S4). To further confirm the results, we performed bisulfite sequencing analysis of the same five gene regions (Supplementary Figure S5). Our data showed that these regions were generally hypomethylated in WT mESCs, in agreement with previous RRBS and WGBS data (48,50), and their methylation levels exhibited either decreases or no changes in *Dnmt3l* KO mESCs (Figure 2F, Supplementary Figure S5).

**Figure 3.**
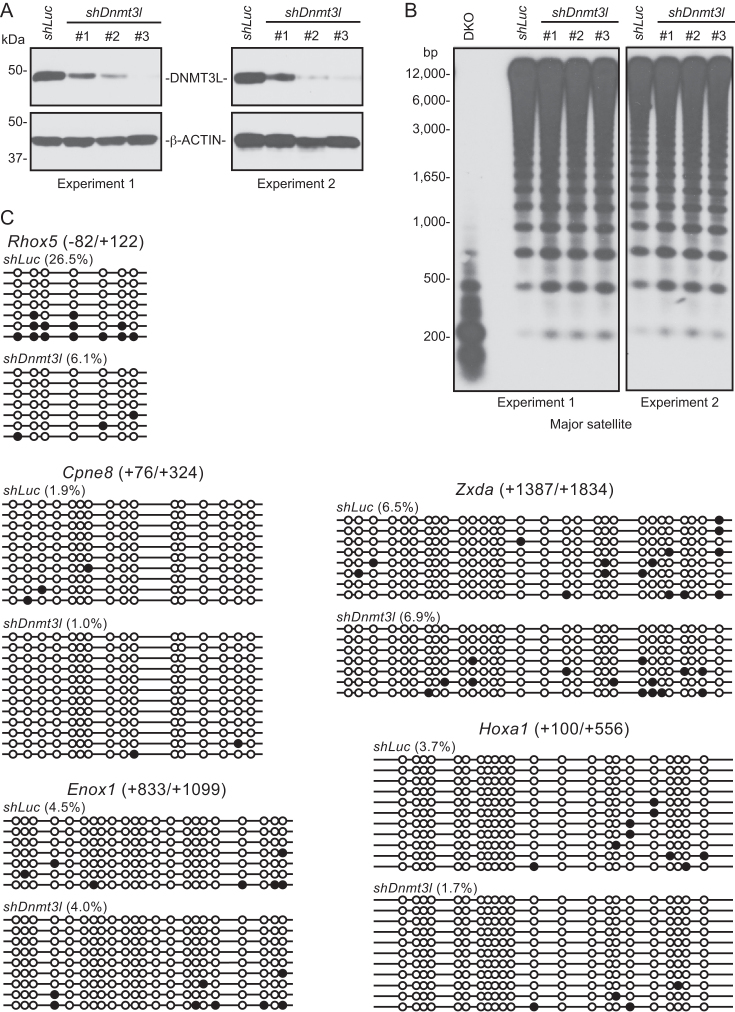
shRNA-induced *Dnmt3l* KD does not lead to gain of methylation. (**A**) Western blot analysis showing that the three *Dnmt3l* shRNAs induced DNMT3L depletion with varying efficiencies (#3>#2>#1). The experiment was performed twice. (**B**) Southern blot analysis showing that *Dnmt3l* KD led to slight decreases of methylation at the major satellite repeats. (**C**) Bisulfite sequencing analysis of the same five gene regions described in Figure 2F, which showed either loss of or no changes in methylation in *Dnmt3l* KD mESCs. Control (*shLuc*) and *Dnmt3l* KD (*shDnmt3l* #3) DNA samples from experiment 1 were used for this experiment.

A key difference between the Neri study and ours was how DNMT3L depletion was achieved. We derived *Dnmt3l* KO mESCs from blastocyst-stage embryos, whereas Neri and colleagues used shRNAs to knock down *Dnmt3l* in an established mESC line. To determine whether the different approaches contributed to the discrepancy in results, we performed KD experiments in the J1 mESC line using the same *Dnmt3l* shRNAs and protocols reported by Neri *et al.* (30). In agreement with their data, all *Dnmt3l* shRNAs substantially reduced DNMT3L levels, with *shDnmt3l* #3 being the most efficient (Figure 3A). Southern blot analysis showed slight loss of methylation at the major satellite repeats in *Dnmt3l* KD mESCs (Figure 3B). The degree of hypomethylation was less than that in *Dnmt3l* KO mESCs (Figure 1D), probably for two reasons: (a) DNMT3L was still present (albeit reduced) in *Dnmt3l* KD mESCs; and (b) these cells were maintained under selection with Puromycin for only three days after transfection of the shRNA plasmids (whereas *Dnmt3l* KO mESCs were cultured for 10–15 passages). Bisulfite sequencing analysis revealed again that the aforementioned five gene regions had low levels of methylation in control (*shLuc*) mESCs and showed either loss of or no changes in methylation in *Dnmt3l* KD mESCs (Figure 3C), contrary to the data of the Neri study that *Dnmt3l* KD led to hypermethylation of these regions (30). While the cause(s) of the discrepancy remain to be determined, one possibility is that the gain of methylation observed by Neri and colleagues was due to partial differentiation of the mESC lines they used. Bivalent promoters are repressed by H3K27me3 and are generally unmethylated or hypomethylated in undifferentiated ESCs. Upon differentiation, some bivalent promoters lose H3K4me3 and become silenced by DNA methylation (48,50).

In summary, our results confirm those of many previous studies that DNMT3L functions as a positive regulator of DNA methylation and provide no evidence that DNMT3L antagonizes DNA methylation in mESCs.

### DNMT3L deficiency mainly affects methylation of DNMT3A target regions

Previous work has demonstrated that the major and minor satellite repeats are preferentially methylated by DNMT3A and DNMT3B, respectively (12). The observation that DNMT3L deficiency resulted in loss of methylation at the major satellite repeats, but not at the minor satellite repeats (Figure 1D, E), led us to hypothesize that DNMT3L preferentially regulates the methylation of DNMT3A target regions.

To test our hypothesis, we sought to compare the DNA methylation profile of *Dnmt3l* KO mESCs with the WGBS data of *Dnmt3a* KO mESCs and *Dnmt3b* KO mESCs that we recently generated (51). For the comparisons, the RRBS data were first converted to 500-bp tiles (qualified tiles were defined as those with ≥3 CpG sites and ≥10-fold coverage) (Supplementary Figure S6A–C). Again, *Dnmt3l* mKO cells had only a small number of hypomethylated tiles, with the majority (22/30, 73%) being in ICRs and showing severe loss of methylation (Supplementary Figure S6A), *Dnmt3l* KO cells had ∼10 times more hypomethylated tiles (331), mostly with moderate loss of methylation (Supplementary Figure S6B), and *Dnmt3l* mzKO showed the combined changes (Supplementary Figure S6C), whereas none of the cell lines had any hypermethylated tiles (Supplementary Figure S6A-C). Analysis of the genomic distribution of the hypomethylated tiles revealed that, in *Dnmt3l* mKO cells, nearly half of them were in promoters, consistent with their enrichment in ICRs, and in *Dnmt3l* KO and *Dnmt3l* mzKO cells, they were mostly located in intergenic regions, gene bodies (introns and exons), and promoters (Supplementary Figure S6D).

**Figure 4.**
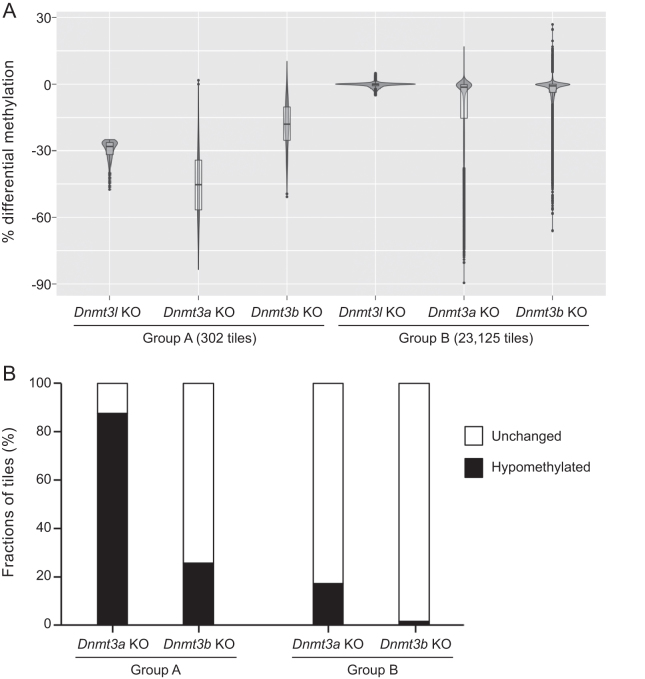
DNMT3L deficiency mainly affects DNMT3A-methylated regions. (**A**) Violin plots of methylation changes using 500-bp tile resolution showing that hypomethylated tiles (methylation loss ≥ 25% and *q*-value ≤ 0.01) in *Dnmt3l* KO (Group A) display more severe hypomethylation in *Dnmt3a* KO mESCs and less severe hypomethylation in *Dnmt3b* KO mESCs, whereas unchanged tiles (methylation difference < 5% and *q*-value > 0.1) in *Dnmt3l* KO mESCs (Group B) are mostly unchanged in *Dnmt3a* KO and *Dnmt3b* KO mESCs. (**B**) Bar graphs showing the fractions (percentages) of hypomethylated and unchanged tiles in *Dnmt3a* KO and *Dnmt3b* KO mESCs (relative to WT mESCs) in Group A and Group B.

We extracted hypomethylated tiles (methylation loss ≥ 25% and *q*-value ≤ 0.01) and unchanged tiles (methylation difference < 5% and *q*-value > 0.1, these criteria were used to select the ‘true’ unchanged tiles by excluding those with methylation loss of 5–25%) in *Dnmt3l* KO mESCs and then compared the DNA methylation changes of the corresponding tiles in *Dnmt3a* KO and *Dnmt3b* KO mESCs relative to WT mESCs. As shown in the violin plots in Figure 4A, the tiles that were hypomethylated in *Dnmt3l* KO cells (Group A) exhibited more severe hypomethylation in *Dnmt3a* KO cells and less severe hypomethylation in *Dnmt3b* KO cells, and the unchanged tiles in *Dnmt3l* KO cells (Group B) were mostly also unchanged in *Dnmt3a* KO and *Dnmt3b* KO cells. Of the 302 hypomethylated tiles in *Dnmt3l* KO cells that were common in *Dnmt3a* KO and *Dnmt3b* KO cells, 265 (∼88%) showed significant hypomethylation in *Dnmt3a* KO cells, whereas only 77 (∼25%) were significantly hypomethylated in *Dnmt3b* KO cells. Of the 23 125 unchanged tiles in *Dnmt3l* KO cells that were common in *Dnmt3a* KO and *Dnmt3b* KO cells, 19 135 (∼83%) in *Dnmt3a* KO and 22 790 (∼99%) in *Dnmt3b* KO cells also showed no significant changes in methylation (Figure 4B). These results suggest that DNMT3L deficiency mainly impairs DNA methylation in DNMT3A target regions in mESCs, in agreement with evidence obtained from genetic studies in mouse that DNMT3L is functionally more critical for DNMT3A than for DNMT3B (13,14,17,28).

### DNMT3A is unstable in the absence of DNMT3L

**Figure 5.**
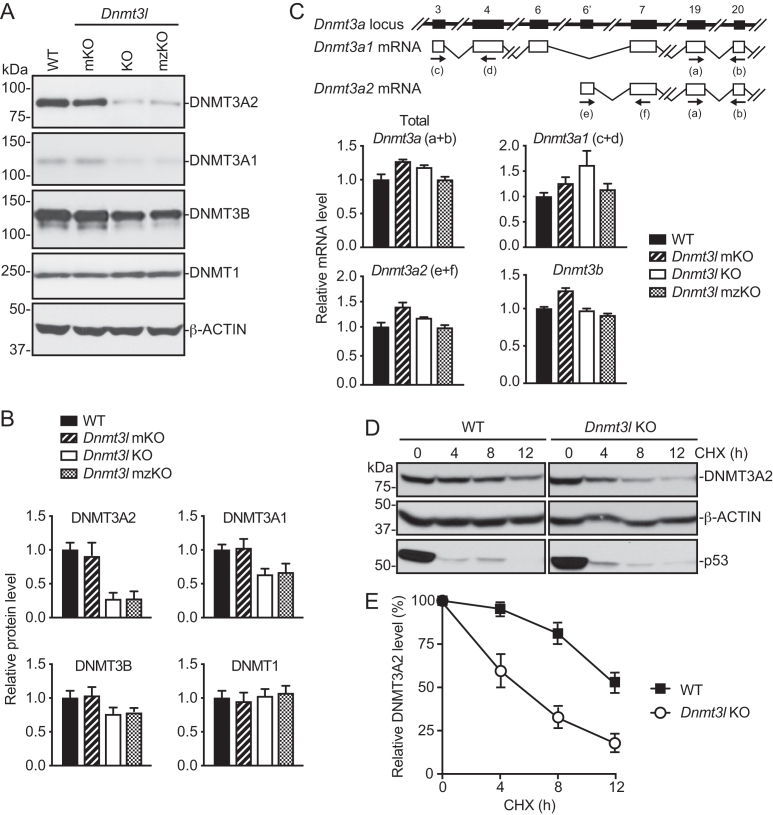
DNMT3A is unstable in the absence of DNMT3L. (**A** and **B**) Western blot analysis of DNMT proteins in DNMT3L-deficient mESCs. Shown are representative blots (A) and quantification of the data (mean + SD from four independent experiments) by densitometry using ImageJ (B). (**C**) RT-qPCR analysis of *Dnmt3a* and *Dnmt3b* mRNAs in DNMT3L-deficient mESCs (mean + SD from two independent experiments). The *Dnmt3a* locus and the two major *Dnmt3a* transcripts, *Dnmt3a1* and *Dnmt3a2*, as well as the locations of the primers (a–f), are schematically shown at the top. (**D** and **E**) Analysis of DNMT3A2 protein stability by inhibiting protein synthesis with cycloheximide (CHX) and then monitoring DNMT3A2 levels by Western blot for different periods of time. p53 was used as a positive control for the effect of CHX treatment, and β-ACTIN was used as a loading control. Shown are representative blots (D) and quantification of the data (mean ± SD from three independent experiments) by densitometry using ImageJ (E).

Biochemical studies indicate that DNMT3L interacts with both DNMT3A and DNMT3B and stimulates their catalytic activities (18–21). Why DNMT3L preferentially regulates DNMT3A-mediated DNA methylation *in vivo* is an open question. To gain insights into the mechanism underlying the functional specificity of DNMT3L, we first examined the expression of the DNMT proteins in *Dnmt3l*-deficient mESCs by Western blot. As compared to WT mESCs, *Dnmt3l* KO and mzKO mESCs, but not *Dnmt3l* mKO mESCs, showed marked (>70%) decreases in DNMT3A2, moderate (∼35–40%) decreases in DNMT3A1 and slight (∼20–25%) decreases in DNMT3B, whereas DNMT1 was expressed at comparable levels in all the cell lines (Figure 5A, B). DNMT3A2 decreases were also observed in shRNA-induced *Dnmt3l* KD mESCs, which were commensurate with *Dnmt3l* KD efficiencies (Supplementary Figure S7). These results suggest that downregulation of DNMT3A, especially DNMT3A2, the predominant isoform in mESCs (52), largely contributed to the preferential effect of DNMT3L deficiency on DNMT3A targets.

Transcription of the two *Dnmt3a* isoforms in mESCs—*Dnmt3a1* (minor isoform) and *Dnmt3a2* (major isoform)—was driven by different promoters (52). RT-qPCR analysis using specific primers revealed that *Dnmt3a1, Dnmt3a2*, total *Dnmt3a*, as well as *Dnmt3b*, mRNA levels were not altered in *Dnmt3l* KO mESC lines (Figure 5C). These data suggest that the decrease in DNMT3A proteins in *Dnmt3l* KO cells was due to dysregulation at the post-transcriptional level.

We next examined whether DNMT3A protein stability is affected in the absence of DNMT3L. To this end, we treated *Dnmt3l* KO and WT mESCs with the protein synthesis inhibitor cycloheximide (CHX) and then monitored the rates of DNMT3A2 decline over time (DNMT3A1 level is too low in mESCs to accurately quantify). p53, a protein that turns over rapidly, was used as a control for the efficacy of CHX treatment, and β-ACTIN, a highly stable protein, served as a loading control. In the absence of DNMT3L, DNMT3A2 declined substantially more rapidly (Figure 5D). By measuring the intensities of the DNMT3A2 bands, we estimated that the half-life of DNMT3A2 protein was reduced to ∼6 hr in *Dnmt3l* KO mESCs, as opposed to more than 12 h in WT mESCs (Figure 5E). These results demonstrate that DNMT3L is required for DNMT3A2 protein stability in mESCs.

Two major pathways—the ubiquitin-proteasome pathway and lysosomal proteolysis—mediate protein degradation in eukaryotic cells. We therefore investigated whether these pathways were involved in DNMT3A2 degradation in the absence of DNMT3L. We treated *Dnmt3l* KO and WT mESCs with the proteasome inhibitor MG132 or the lysosome inhibitor Bafilomycin A1 (Baf-A1) for different periods of time and then examined DNMT3A2 levels by Western blot analysis. Although the inhibitors were highly effective, as evidenced by the dramatic accumulation of p53 and LC3B-II, respectively, they failed to rescue DNMT3A2 levels in *Dnmt3l* KO mESCs (Supplementary Figure S8A, B). These results suggest that a mechanism independent of the ubiquitin-proteosome and lysosomal proteolysis pathways is responsible for DNMT3A2 degradation in DNMT3L-deficient mESCs.

### DNMT3L-DNMT3A2 complex formation is critical for DNMT3A2 stability

**Figure 6.**
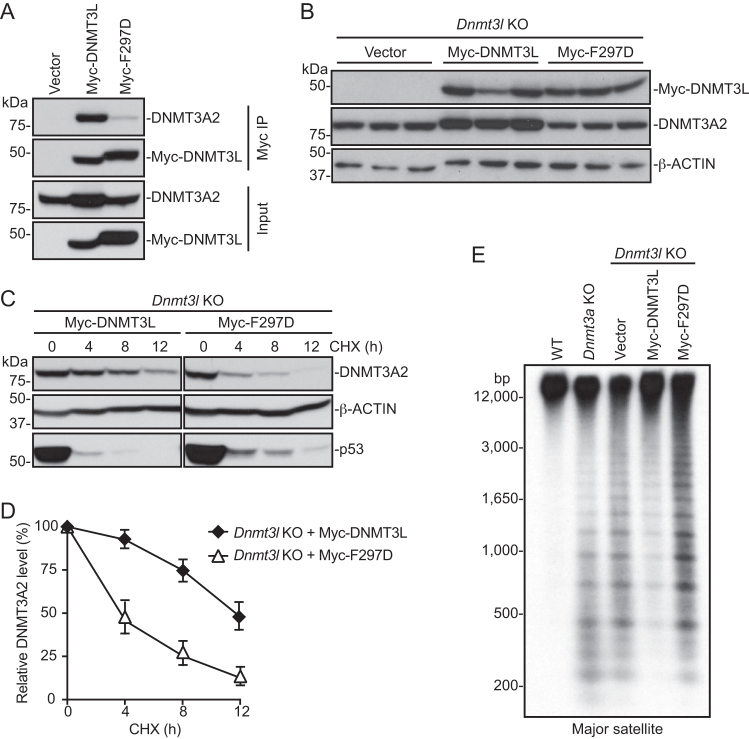
The ability of DNMT3L to interact with DNMT3A2 is critical for DNMT3A2 stability. (**A**) Co-immunoprecipitation experiment showing that Myc-tagged WT DNMT3L, but not the F297D mutant, interacts with endogenous DNMT3A2. (**B**) Western blot analysis showing that stable expression of WT DNMT3L, but not the F297D mutant, rescues DNMT3A2 levels in *Dnmt3l* KO mESCs. Different lanes represent independent clones. (**C** and **D**) Analysis of DNMT3A2 protein stability showing that WT DNMT3L, but not the F297D mutant, restores DNMT3A2 stability. Shown are representative blots (C) and quantification of the data (mean ± SD from three independent experiments) by densitometry using ImageJ (D). (**E**) Southern blot analysis showing that expression of WT DNMT3L, but not the F297D mutant, in *Dnmt3l* KO mESCs results in recovery of DNA methylation at the major satellite repeats.

Biochemical and structural studies have shown that DNMT3L and DNMT3A directly interact via their C-terminal regions (24–26). We therefore hypothesized that formation of the DNMT3L-DNMT3A2 complex could lead to DNMT3A2 stabilization. To test the hypothesis, we first sought to engineer a DNMT3L point mutation that would abolish DNMT3L-DNMT3A interaction by substituting phenylalanine 297 of mouse DNMT3L with aspartate (F297D). F297 (equivalent to F261 in human DNMT3L) is a critical residue involved in interacting with DNMT3A, and replacing this hydrophobic amino acid with the negatively charged aspartate has been shown to abolish the ability of DNMT3L to stimulate DNMT3A catalytic activity (24,26). By co-immunoprecipitation (co-IP) experiments using Myc-tagged DNMT3L proteins transiently expressed in mESCs, we confirmed that WT DNMT3L interacted with endogenous DNMT3A2 and that the F297D mutation largely disrupted the interaction (Figure 6A). We then performed rescue experiments to compare the effects of WT DNMT3L and the F297D mutant in restoring DNMT3A2 levels in *Dnmt3l* KO mESCs. To this end, Myc-tagged DNMT3L or the F297D mutant was transfected in *Dnmt3l* KO mESCs, and individual stable clones were obtained after 7–10 days of selection with blasticidin. Expression of WT DNMT3L, but not the F297D mutant, resulted in obvious restoration of DNMT3A2 levels, as compared to stable clones transfected with the empty vector (Figure 6B). To verify that formation of the DNMT3L-DNMT3A2 complex led to DNMT3A2 stabilization, we again assessed DNMT3A2 stability, as described above (Figure 5D, E), in the stable clones reconstituted with Myc-tagged DNMT3L proteins. Indeed, DNMT3A2 stability almost completely recovered in cells expressing WT DNMT3L (half-life: ∼12 h), and the effect was not observed in cells expressing the F297D mutant (Figure 6C, D). Southern blot analysis also confirmed that the DNA methylation level at the major satellite repeats was rescued to a large extent in *Dnmt3l* KO mESCs reconstituted with WT DNMT3L, whereas expression of the F297D mutant protein had no effect (Figure 6E). Taken together, these results support our hypothesis that DNMT3L, by interacting with DNMT3A2 to form a complex, prevents DNMT3A2 from being degraded.

### Restoring DNMT3A level in *Dnmt3l* KO mESCs partially recovers DNA methylation

**Figure 7.**
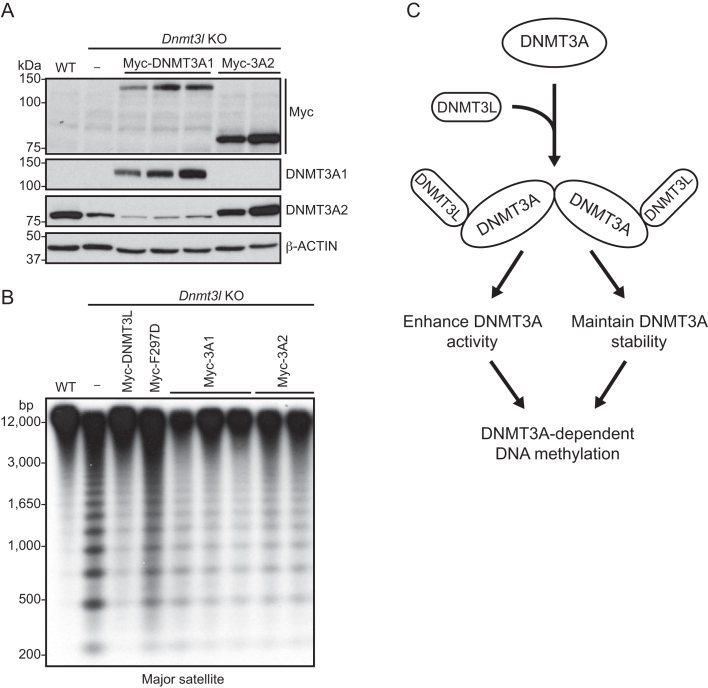
Restoring DNMT3A levels in *Dnmt3l* KO mESCs partially rescues DNA methylation. (**A**) Western blots with Myc and DNMT3A antibodies showing the expression of Myc-DNMT3A1 or Myc-DNMT3A2 in stable clones generated in *Dnmt3l* KO mESCs. Different lanes represent independent clones. (**B**) Southern blot analysis showing partial recovery of DNA methylation at the major satellite repeats in Myc-DNMT3A1 or Myc-DNMT3A2 stable clones generated in *Dnmt3l* KO mESCs. (**C**) Proposed model suggesting that the formation of the DNMT3L–DNMT3A complex (heterotetramers) results in stimulation of DNMT3A activity and maintenance of DNMT3A stability, both contributing to the role of DNMT3L in facilitating DNA methylation.

Our data indicated that DNMT3L plays a crucial role in maintaining DNMT3A protein stability, in addition to its known function in stimulating DNMT3A catalytic activity (18,19,21). To determine the relative contributions of the two regulatory effects of DNMT3L in DNMT3A-mediated DNA methylation, we asked whether restoring the DNMT3A level in the absence of DNMT3L would be sufficient to rescue DNA methylation. We generated stable clones in *Dnmt3l* KO mESCs expressing Myc-tagged DNMT3A1 or DNMT3A2. We selected clones with protein levels close to the endogenous DNMT3A2 level in WT mESCs for experiments (Figure 7A). Southern blot analysis revealed that expression of either DNMT3A1 or DNMT3A2 in *Dnmt3l* KO mESCs substantially restored the methylation levels at the major satellite repeats, although the rescue efficiency of DNMT3A proteins was not as high as that of DNMT3L (Figure 7B). This observation, together with previous *in vitro* studies showing that the interaction of DNMT3L increases the catalytic efficiency of DNMT3A enzyme (21), led us to conclude that maintaining DNMT3A protein stability is an important aspect of DNMT3L function and that the regulatory roles of DNMT3L in DNMT3A abundance and catalytic activity both contribute to DNMT3A-dependent DNA methylation *in vivo* (Figure 7C).

## DISCUSSION

DNMT3L is a key regulator of *de novo* DNA methylation. Based on its ability to stimulate the enzymatic activities of DNMT3A and DNMT3B *in vitro* (18,19,21), it is generally assumed that DNMT3L functions primarily as a cofactor of these enzymes. In this study, we demonstrate that, in mESCs, DNMT3L also regulates the abundance of these *de novo* methyltransferases, especially DNMT3A2, the predominant DNMT3A protein product in mESCs (52). Our results indicate that DNMT3L, via interacting with DNMT3A2 to form a complex, prevents DNMT3A2 from being degraded. DNMT3L-deficient mESCs show hypomethylation at genomic regions that are mainly DNMT3A targets, including the major satellite repeats. Restoring DNMT3A amount (by forced expression of either DNMT3A1 or DNMT3A2) in DNMT3L-deficient mESCs partially, but significantly, rescues DNA methylation levels at the major satellite repeats. Therefore, we conclude that the regulatory roles of DNMT3L in DNMT3A activity and stability both contribute to DNMT3A-dependent methylation and functions (Figure 7C).

During the mammalian life cycle, two waves of *de novo* DNA methylation take place—the first occurring shortly after implantation, which establishes the initial methylation pattern in the embryo, and the second occurring during germ cell maturation, which establishes germ line-specific methylation marks, including genomic imprints (1,2). Genetic studies and genome-wide DNA methylation analysis suggest that DNMT3B plays a major role in *de novo* methylation during embryogenesis (first wave) and DNMT3A is largely responsible for *de novo* methylation in germ cells (second wave) (13,14,42,43,53). DNMT3L is essential for DNMT3A-mediated methylation in germ cells but appears to be dispensable for DNMT3B-mediated methylation and functions during embryonic development (17,28,29,42,43). Our finding that DNMT3L preferentially stabilizes DNMT3A, especially DNMT3A2, in mESCs provides a plausible explanation for the functional specificity of DNMT3L *in vivo*. Specifically, *Dnmt3l* KO and *Dnmt3a* KO mice exhibit almost identical germline phenotypes (14,17,28). Both DNMT3A2 and DNMT3L are highly expressed in prospermatogonia and growing oocytes (54,55), the stages when active *de novo* DNA methylation occurs during gametogenesis. It will be interesting to determine whether DNMT3L is important for maintaining DNMT3A2 stability in the germline.

While the zygotic function of DNMT3L is not required for mammalian development (17,28), our results suggest that it may be involved in the regulation of DNA methylation at a subset of genomic regions during early embryogenesis. However, previous work has shown that DNA methylation subsequently ‘catches up’ in *Dnmt3l* KO embryos due to upregulation of *Dnmt3a* and *Dnmt3b* transcription (56). Indeed, upon cellular differentiation, *Dnmt3l* is quickly silenced, and *Dnmt3a* and *Dnmt3b* transcription is transiently upregulated and then maintained at low levels in differentiated cell types. Our data indicate that DNMT3L does not regulate *Dnmt3a* and *Dnmt3b* transcription in mESCs. It remains to be determined whether DNMT3L deficiency affects *Dnmt3a* and/or *Dnmt3b* transcription during cellular differentiation. We attempted to address this issue by performing the EB formation assay but found that *Dnmt3l* KO mESCs exhibited obvious differentiation defects. While this observation was in agreement with previous findings that DNA hypomethylation impairs mESC differentiation (31,46), it was counterintuitive, given that *Dnmt3l* KO mice exhibit no obvious developmental phenotype (17,28). It is possible that the *Dnmt3l* KO mESCs we used (which had been cultured for 10–15 passages) had more severe hypomethylation than *Dnmt3l* KO embryos and/or that EB formation cannot recapitulate the mechanisms that recover DNA methylation deficiencies during development in utero.

It remains to be determined why DNMT3L deficiency has a more severe effect on DNMT3A2 stability than on DNMT3A1 and DNMT3B levels. While recombinant or exogenously expressed DNMT3A, DNMT3B and DNMT3L proteins are capable of interacting with each other (19,21–23), there is evidence that endogenous DNMT3L physically interacts with DNMT3A2, but not with DNMT3A1 and DNMT3B, in mESCs (57). This provides a possible explanation for the preferential effect of DNMT3L on DNMT3A2 stability. The full-length DNMT3A1 and DNMT3B proteins have high sequence homology except a variable region at their N termini, and this region is absent in the shorter DNMT3A2 isoform (52). However, this variable region is unlikely to be directly involved in interacting with DNMT3L, as structural evidence indicates that the DNMT3L-DNMT3A2 interaction is mediated by their C-terminal regions (24,25). One possibility that endogenous DNMT3A1 and DNMT3B fail to form complexes with DNMT3L in mESCs is that they are less accessible to DNMT3L. DNMT3A1 and DNMT3B are tightly associated with chromatin, especially heterochromatin, whereas DNMT3A2 seems to be more loosely bound to chromatin (52). That DNMT3A2 is relatively more soluble and more widely distributed perhaps makes it more susceptible to degradation in the absence of DNMT3L. Alternatively, the extra sequences in the full-length DNMT3A1 and DNMT3B proteins may protect them from being degraded. Another factor that needs to be considered is that, in mESCs, DNMT3A2 is far more abundant than DNMT3A1 (52), which may make DNMT3A2 more sensitive to the effects of DNMT3L deficiency. DNMT3B is also abundantly expressed in mESCs. However, *Dnmt3b* produces multiple alternatively spliced isoforms, some of which have no catalytic activity but may play regulatory roles in DNMT3B-mediated methylation (58), similar to the effects of DNMT3L on DNMT3A. While the mechanism by which DNMT3A2 is degraded remains to be elucidated, our data suggest that it is likely independent of the ubiquitin–proteasome and lysosomal proteolysis pathways. Previous work has shown that DNMT3L converts DNMT3A from heterogeneous large homomultimers into a defined, soluble heterotetramer, because it lacks one of the protein-protein interaction interfaces present in DNMT3A (59). In the absence of DNMT3L, aggregated DNMT3A may be prone to degradation.

In agreement with previous studies in germ cells, embryos and mESCs (17,28,29,42,43,45,56,57,60), our results show that DNMT3L is a positive regulator of DNA methylation. The quality of our mESC lines and RRBS datasets was validated by expected patterns in methylation at ICRs, which are major DNMT3L targets. Specifically, maternal ICRs showed ∼50% methylation in WT mESCs (consistent with monoallelic methylation), severe hypomethylation in *Dnmt3l* mKO and mzKO mESCs, and complete resistance to hypomethylation in *Dnmt3l* KO mESCs. Our RRBS analysis showed no evidence of gain of methylation in DNMT3L-deficient mESCs reported by Neri *et al.* (30). The conclusion of the Neri study was based on genome-wide MeDIP-seq data and bisulfite sequencing analysis of individual loci in shRNA-induced *Dnmt3l* KD mESCs. However, their MeDIP-seq analysis had serious flaws. Most notably, the lack of replicates, both biological and technical, would hamper normalization and comparison of samples based on statistical power. Indeed, we re-analyzed their MeDIP-seq data. A striking outcome of these analyses was that the frequency of hypomethylated and hypermethylated counts varied significantly depending on the methods of normalization, blurring the conclusion to what extent hypermethylation is actually present. Thus, confidence in any conclusion derived from that dataset is unwarranted. Also, our bisulfite sequencing analysis of both *Dnmt3l* KO and shRNA-induced *Dnmt3l* KD mESCs could not reproduce the gain of methylation at the five bivalent promoter regions verified by Neri *et al.* (30). Differences in the methods of DNA methylation analysis and the mESC lines may have contributed to the discrepancy between the two studies. As capture of DNA by MeDIP is highly influenced by local CpG density, and so is the efficiency of amplification during library preparation, MeDIP-seq is being replaced by more accurate, bisulfite conversion-based methods, such as RRBS and WGBS. ESCs are prone to differentiation, which results in gain of methylation at some genomic regions, including bivalent promoters (48). We noticed that even the control (shGFP) mESC line used in the Neri study had universally higher methylation levels at the five bivalent promoters than all the mESC lines (WT, *Dnmt3l* KO, *shLuc* and *shDnmt3l*) we used. More troubling was the observation by Neri *et al.* that their *Dnmt3l* KD mESCs exhibited morphological alterations (30), which we did not observe. Another difference was that, unlike our *Dnmt3l* KD and *Dnmt3l* KO mESC lines that exhibited decreased DNMT3A2 levels, Neri *et al.* showed no changes in the protein level of DNMT3A (the band shown was likely DNMT3A2 based on the signal intensity) in their *Dnmt3l* KD cells (30). However, from their microarray data, *Dnmt3a1* and *Dnmt3a2* transcripts were undetectable in all samples, and *Dnmt3b* and *Pou5f1* (encoding OCT4) transcripts were also substantially decreased in the sample with the highest *Dnmt3l* KD efficiency (shRNA #3). *Dnmt3a2, Dnmt3b* and *Pou5f1* are supposed to be highly expressed in undifferentiated mESCs and become downregulated during differentiation. Based on these indications, it is highly likely that the gain of methylation observed by Neri and colleagues was an artifact of partial differentiation of the mESC lines they used.

In summary, we demonstrate in this study that DNMT3L is important for DNMT3A stability, in addition to its role in regulating DNMT3A catalytic activity. Our finding helps better understand the functional specificity of DNMT3L *in vivo* (i.e. regulating DNMT3A-dependent methylation and functions, such as the establishment of genomic imprints in germ cells).

## DATA AVAILABILITY

The RRBS data has been deposited in the GEO database (accession number GSE116489).

## Supplementary Material

Supplementary Data
