# Supplementary material for: DNMT3L facilitates DNA methylation partly by maintaining DNMT3A stability in mouse embryonic stem cells
Source: Nucleic Acids Res. 2018 Oct 13;47(1):152–67. doi: 10.1093/nar/gky947 (PMC6326784; doi:10.1093/nar/gky947)
Supplement: Supplementary Data [file gky947_supplemental_files.zip › Supplementary Data.pdf]

Supplementary Table S1. Primers and oligonucleotides used in the study

Application	Target	Sequence (5' to 3')	Reference
Cloning	Dnmt3L (F)	AGGGAATTCCCGGGAGACACCTTCTTC	This study
Cloning	Dnmt3L (R)	TCAGAATTCTAAAGAGGAAGTGAGTTTTG	This study
Cloning	Dnmt3L F297D (F)*	CTGGTACATGgaCCAGTTCCACCGGATCCT	This study
Cloning	Dnmt3L F297D (R)*	GTGGAACTGGtcCATGTACCAGCCGGGACA	This study
Cloning	Dnmt3a1 (F)	CCTGAATTCGCCCTCCAGCGGCCCCGG	This study
Cloning	Dnmt3a2 (F)	TGCGAATTCGAATGCTGTGGAAGAGAAC	This study
Cloning	Dnmt3a (R)	CACGAATTCAGTTTGCCCCCATGTCCCT	This study
Southern blot	Major satellite probe	TTAGAAATGTCCACTGTAGGACGTGGAATATGGCAAG	12
Southern blot	Minor satellite probe	ACTGAAAAACACATTCGTTGGAAACGGGATTTGTAGA ACAGTGTATATCAATGAGTTACAATGAG	33
Genotyping	Sry (F)	TCTTAAACTCTGAAGAAGAGAC	This study
Genotyping	Sry (R)	GTCTTGCCTGTATGTGATGG	This study
Genotyping	Dnmt3L (F)	CTGTGAGAGCCCCGACTGTACCAG	This study
Genotyping	Dnmt3L (R)	CAGAGGGAATGGCTGAATGGTGGC	This study
Genotyping	Neomycin^{r} (F)	ATGGGATCGGCCATTGAACAAG	This study
Genotyping	Neomycin^{r} (R)	CATCGCCATGGGTCACGACGAGATC	This study
Bisulfite seq	Cpne8 (F)	GTAGTTTGGATGTGGGTT	This study
Bisulfite seq	Cpne8 (R)	CAACATACAACTTCTCACCAA	This study
Bisulfite seq	Rhox5 (F)	TAGTAAAGAAGTTATGGGTTAATTG	This study
Bisulfite seq	Rhox5 (R)	ATCCCTACTAAACATTCCTAACCAC	This study
Bisulfite seq	Enox1 (F)	TTGGGAATTGGATATAAGGATAGG	30
Bisulfite seq	Enox1 (R)	TTATAAAATCAACCAATCAAAACCC	30
Bisulfite seq	Hoxa1 (F)	GATAATGTAAGAATGAATTTTTTTT	30
Bisulfite seq	Hoxa1 (R)	TACCCCCAACATAACCCTAATAATA	30
Bisulfite seq	Zxda (F)	GAGTAGGAGAATTTTTTTAAATG	30
Bisulfite seq	Zxda (R)	AAATTATACTATAACCTTTCAAATACTC	30
RT-qPCR	Dnmt3a (F)	GTTCTACCGCCTCCTGCATGATGC	This study
RT-qPCR	Dnmt3a (R)	GCCCTGTGTGCAGCAGACACTTC	This study
RT-qPCR	Dnmt3a1 (F)	GAGGCCTGGCCGGAAGCGCAAGCAC	This study
RT-qPCR	Dnmt3a1 (R)	GTCTCAGTTCCCTCTCCTTCAGCTG	This study
RT-qPCR	Dnmt3a2 (F)	GAGGGGCTGCACCTGGCCTTATG	This study
RT-qPCR	Dnmt3a2 (R)	AGCATCCCCTCCTACTGGCTCAG	This study
RT-qPCR	Dnmt3b (F)	GGAGGCCCATTAGAGTCCTGTCTC	This study
RT-qPCR	Dnmt3b (R)	CACCAATCACCAAGTCGAACGGGC	This study
RT-qPCR	GAPDH (F)	AAGAGAGGCCCTATCCCAACTC	This study
RT-qPCR	GAPDH (R)	TTGTGGGTGCAGCGAACTTTATTG	This study

Bisulfite seq, Bisulfite sequencing; F, forward primer; R, reverse primer. EcoRI site used for cloning is underlined. *Primers used together with Dnmt3L (F) and Dnmt3L (R) to generate the Dnmt3L F297D mutation (altered nucleotides in lower case).

Supplementary Table S2. Antibodies used in the study

Application	Antibody	Dilution	Vendor & Catalogue #
WB	DNMT3L	1:1,000	Cell Signaling 12309
WB	DNMT3A	1:4,000	Abcam 13888
WB	DNMT3B	1:2,000	Abcam 13604
WB	DNMT1	1:1,000	Cell Signaling 5032
WB	p53	1:1,000	Cell Signaling 2524
WB	LC3B	1:1,000	Cell Signaling 2775
WB	OCT4	1:1,000	Abcam 19857
WB	SOX2	1:1,000	Abcam 97959
WB	β-ACTIN	1:5,000	Sigma A5441
WB	Myc tag	1:5,000	Cell Signaling 2278
IP	Myc tag	1:5,000	Sigma M4439
IF	5mC	1:2,000	Millipore MABE146
IF	5hmC	1:1,000	Active Motif 39769

WB, Western blot; IP, immunoprecipitation; IF, immunofluorescence

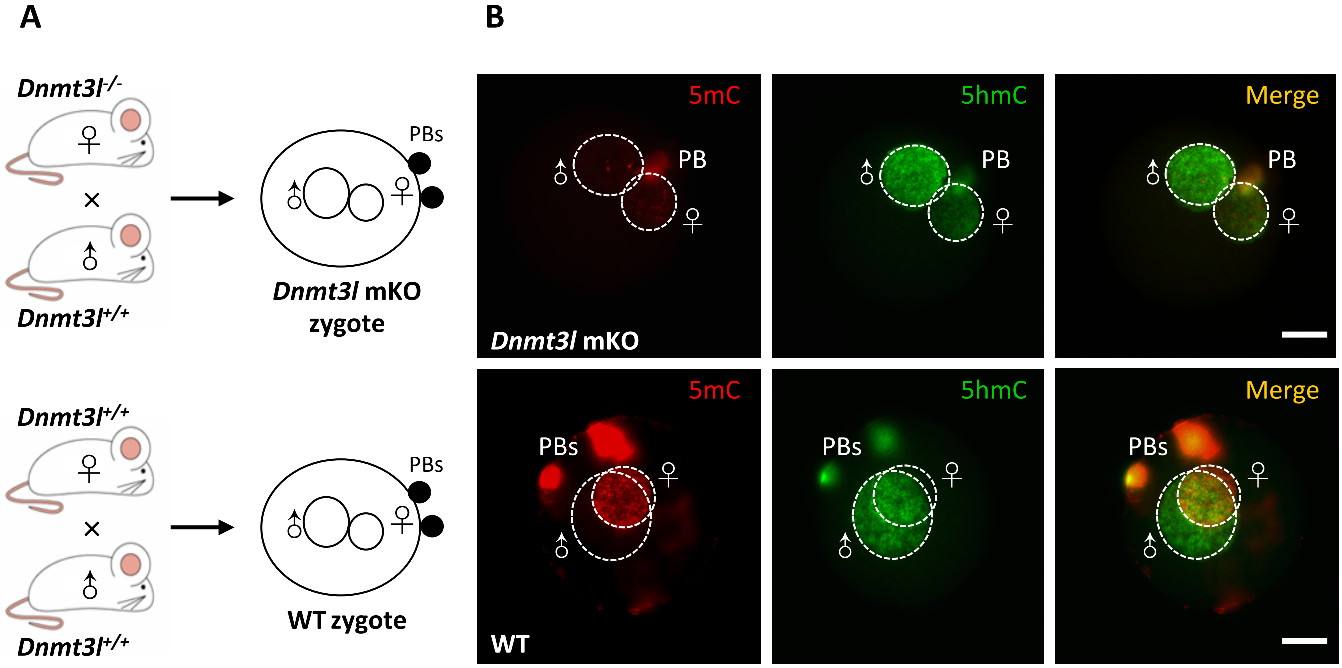

Supplementary Figure S1. Zygotes derived from *Dnmt3l*$^{-/-}$ female mice show global DNA hypomethylation. (**A**) Mouse breeding strategies to obtain *Dnmt3l* mKO and WT zygotes. (**B**) 5mC and 5hmC staining showing that, in a *Dnmt3l* mKO zygote, the female pronucleus is globally hypomethylated and the male pronucleus undergoes 5mC-to-5hmC transition normally (compared to a WT zygote). The male and female pronuclei, as well as polar bodies (PBs), are indicated. Scale bars, 50 μM.

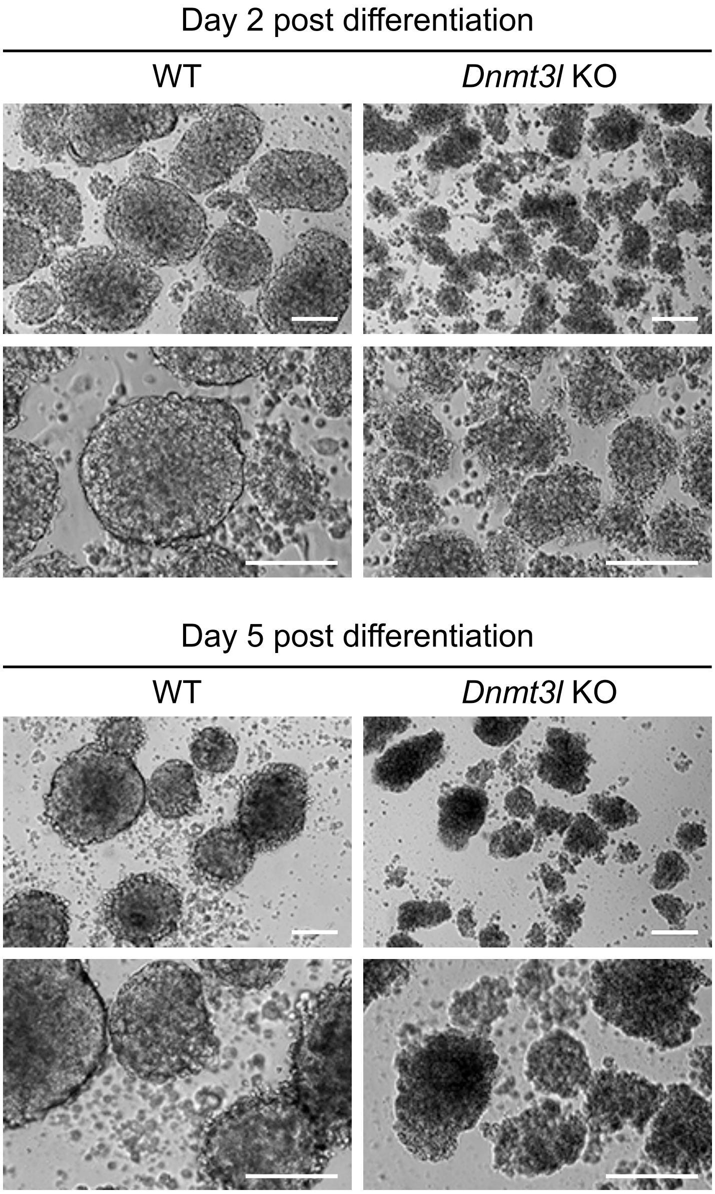

Supplementary Figure S2. *Dnmt3l* KO mESCs show differentiation defects.
WT and *Dnmt3l* KO mESCs were differentiated for five days. Shown are representative images of embryoid bodies (EBs) at day 2 and day 5 post differentiation. Scale bars, 100 μm.

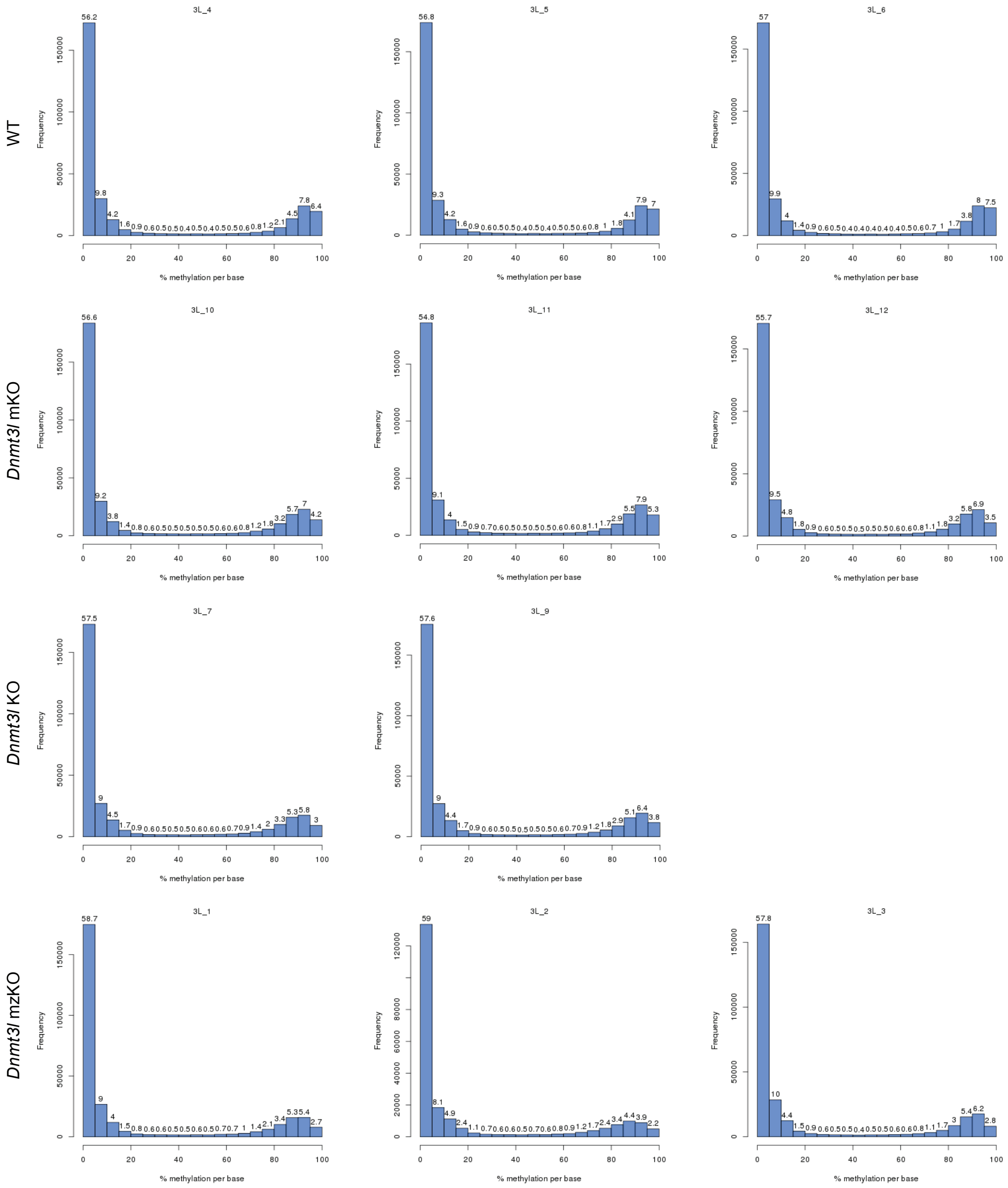

Supplementary Figure S3. Bimodal distribution of CpG methylation in all samples.
Histograms representing the frequency and levels of CpG methylation in percentage per base from the raw data of the three different biological replicates (mESCs clones) of each of the *Dnmt3l* genotype (i.e. WT, *Dnmt3l* mKO, KO and mzKO) that were analyzed by RRBS (one *Dnmt3l* KO sample was not included due to poor quality).

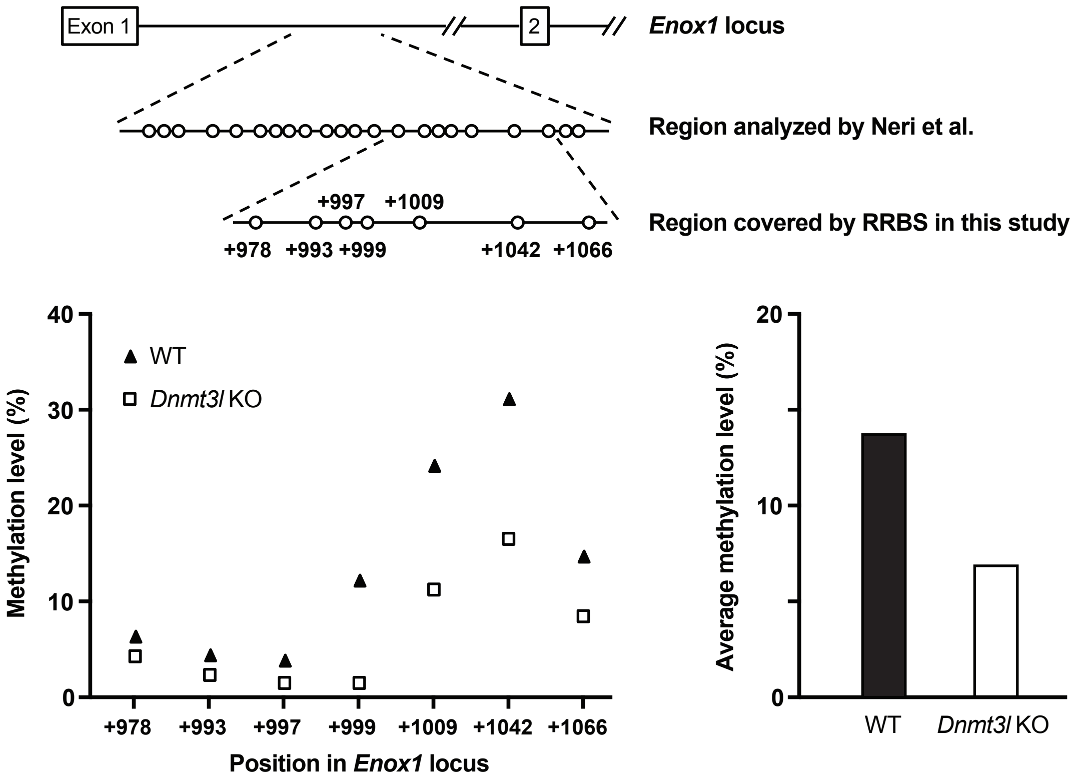

Supplementary Figure S4. Loss of methylation at several CpG sites in the *Enox1* locus in *Dnmt3l* KO mESCs. The *Enox1* locus, the region examined by Neri and colleagues with bisulfite sequencing, and the region with sufficient coverage in the RRBS analysis of the present study are schematically shown at the top. Shown at the bottom are RRBS results indicating the methylation level of each of the seven CpG sites (left) and the average methylation levels of these sites (right) in WT and *Dnmt3l* mESCs. The positions of the Cs of the CpG sites, relative to TSS, are indicated.

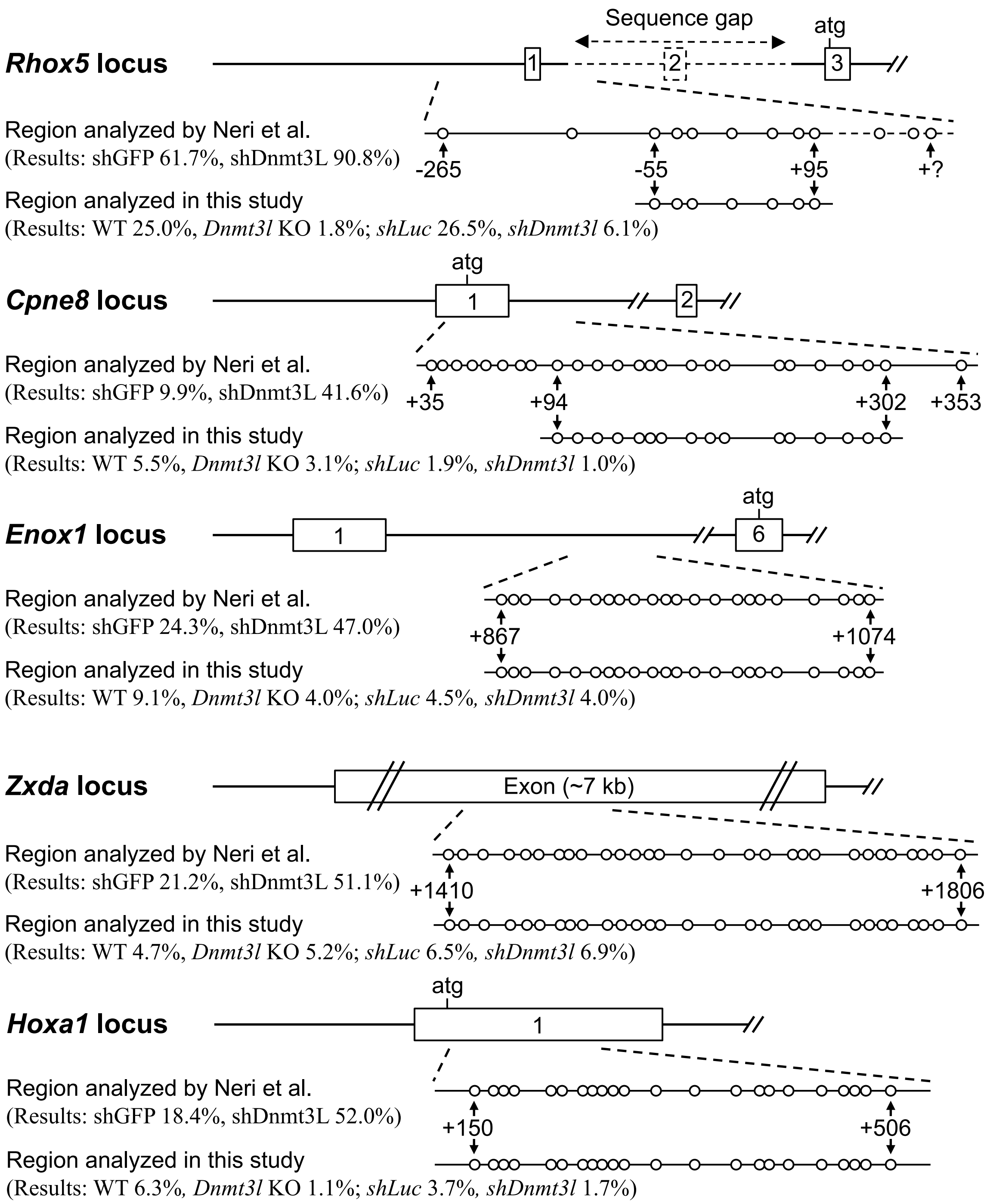

Supplementary Figure S5. Gene regions analyzed by bisulfite sequencing.
The five gene loci and the regions analyzed by bisulfite sequencing are schematically shown. For each gene, the first exon and the exon containing the translation initiation codon (atg) are shown (*Zxda* has a single exon that encodes a long non-coding RNA). For each region analyzed, the locations of the first and last CpG sites (relative to TSS) are indicated (the regions analyzed by Neri et al. were determined from the reported primer information, except the *Rhox5* region, whose 3' portion, including three CpG sites, could not be definitively determined due to a sequence gap in the public database. As the *Rhox5* and *Cpne8* primers described by Neri et al. showed low amplification efficiency, we amplified smaller fragments that contained most of the CpG sites analyzed by Neri and colleagues. For comparison, the results (methylation levels, in percentages) of this study and those reported by Neri et al. are indicated.

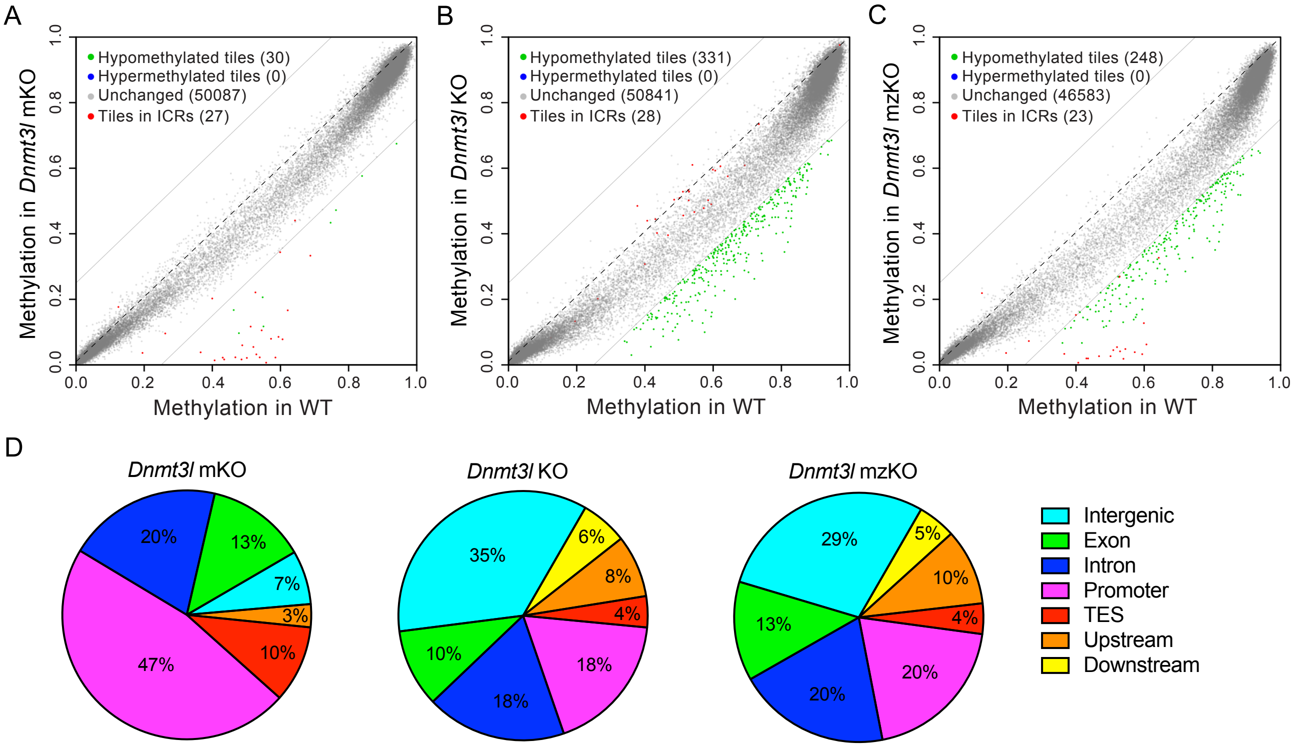

Supplementary Figure S6. Distribution of hypomethylated regions in the genome in DNMT3L-deficient mESCs. (**A** to **C**) The RRBS data shown in Figure 2A-C were converted to 500-bp tiles. (**D**) Pie charts showing the distributions of hypomethylated tiles in different genomic regions in *Dnmt3l* mKO, *Dnmt3l* KO, and *Dnmt3l* mzKO mESCs.

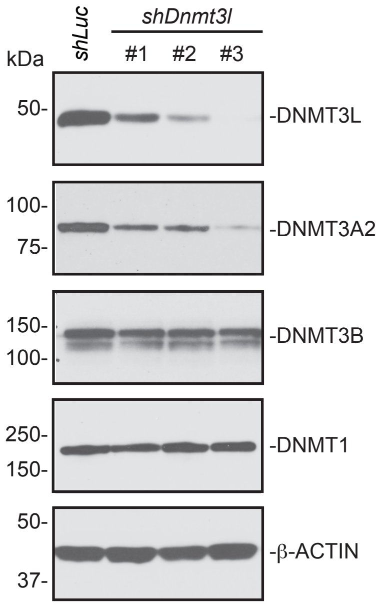

Supplementary Figure S7. *Dnmt3l* KD mESCs show decreased levels of DNMT3A2.
Western blot analysis showing the levels of DNMT3A2, DNMT3B, and DNMT1 in control (*shLuc*) and *Dnmt3l* KD (*shDnmt3l* #1, #2, #3) mESCs.

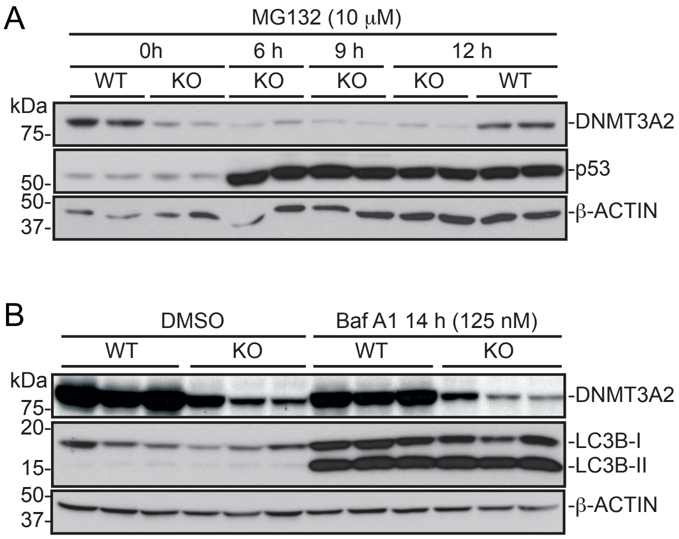

Supplementary Figure S8. Proteosome or lysosome inhibitor fails to rescue DNMT3A2 level in *Dnmt3l* KO mESCs. (**A** and **B**) Western blot results showing that inhibition of the proteosomal or lysosomal protein degradation pathway with MG132 (**A**) or Baf-A1 (**B**) has no effect on DNMT3A2 level in *Dnmt3l* KO mESCs. p53 and LC3B were used as positive controls for MG132 and Baf-A1 treatment, respectively, and β-ACTIN served as a loading control. For each genotype, 2 or 3 independent cell lines were used for the experiments.
